# Supplementary material for: Profiling of the most reliable mutations from sequenced SARS-CoV-2 genomes scattered in Uzbekistan
Source: PLoS One. 2022 Mar 31;17(3):e0266417. doi: 10.1371/journal.pone.0266417 (PMC8970392; doi:10.1371/journal.pone.0266417)
Supplement: S2 Table — (DOCX) [file pone.0266417.s003.docx]

**S2 Table: Genomes representing each of the major evolutionary lineages represented in our cohort.**

| **#** | **Sample ID** | **GISAID ID** | **Virus name** | **Collection date** | **Lineage (GISAID Clade)** | **Originating laboratory** |
| --- | --- | --- | --- | --- | --- | --- |
| **1** | **1** | EPI_ISL_1402423 | hCoV-19/Uzbekistan/Tashkent-CGB-01/2021 | 2020-10-14 | B.1.1.317 (GR) | Private clinic of BiogenMed, Tashkent, Uzbekistan |
| **2** | **2** | EPI_ISL_1477044 | hCoV-19/Uzbekistan/Tashkent-CGB-02/2020 | 2020-10-14 | B.1.1 (GR) | Private clinic of BiogenMed, Tashkent, Uzbekistan |
| **3** | **3** | EPI_ISL_1439578 | hCoV-19/Uzbekistan/Tashkent-CGB-03/2021 | 2020-10-14 | A.24 (S) | Private clinic of BiogenMed, Tashkent, Uzbekistan |
| **4** | **4** | EPI_ISL_1402424 | hCoV-19/Uzbekistan/Tashkent-CGB-04/2021 | 2020-10-14 | B.1.1 (GR) | Private clinic of BiogenMed, Tashkent, Uzbekistan |
| **5** | **5** | EPI_ISL_1402425 | hCoV-19/Uzbekistan/Tashkent-CGB-05/2021 | 2020-10-14 | B.1.1 (GR) | Private clinic of BiogenMed, Tashkent, Uzbekistan |
| **6** | **6** | EPI_ISL_1402426 | hCoV-19/Uzbekistan/Tashkent-CGB-06/2021 | 2020-10-14 | A (S) | Private clinic of BiogenMed, Tashkent, Uzbekistan |
| **7** | **7** | EPI_ISL_1439580 | hCoV-19/Uzbekistan/Tashkent-CGB-07/2021 | 2020-10-14 | B.1.1 (GR) | Private clinic of BiogenMed, Tashkent, Uzbekistan |
| **8** | **8** | EPI_ISL_1402427 | hCoV-19/Uzbekistan/Tashkent-CGB-08/2021 | 2020-10-14 | B.1.1.294 (GR) | Private clinic of BiogenMed, Tashkent, Uzbekistan |
| **9** | **10** | EPI_ISL_1477045 | hCoV-19/Uzbekistan/Tashkent-CGB-10/2020 | 2020-10-14 | B.1.1.274 (GR) | Private clinic of BiogenMed, Tashkent, Uzbekistan |
| **10** | **11** | EPI_ISL_1402428 | hCoV-19/Uzbekistan/Tashkent-CGB-11/2021 | 2020-10-14 | B.1.1.294 (GR) | Private clinic of BiogenMed, Tashkent, Uzbekistan |
| **11** | **12** | EPI_ISL_1439584 | hCoV-19/Uzbekistan/Tashkent-CGB-12/2021 | 2020-10-14 | B.1.1 (GR) | Private clinic of BiogenMed, Tashkent, Uzbekistan |
| **12** | **13** | EPI_ISL_1477046 | hCoV-19/Uzbekistan/Tashkent-CGB-13/2020 | 2020-10-14 | B.1.1.294 (GR) | Private clinic of BiogenMed, Tashkent, Uzbekistan |
| **13** | **14** | EPI_ISL_1439589 | hCoV-19/Uzbekistan/Tashkent-CGB-14/2021 | 2020-10-14 | B.1.1 (GR) | Private clinic of BiogenMed, Tashkent, Uzbekistan |
| **14** | **15** | EPI_ISL_1439593 | hCoV-19/Uzbekistan/Tashkent-CGB-15/2021 | 2020-10-14 | B.1.1.274 (GR) | Private clinic of BiogenMed, Tashkent, Uzbekistan |
| **15** | **17** | EPI_ISL_1477047 | hCoV-19/Uzbekistan/Tashkent-CGB-17/2020 | 2020-12-07 | B.1.1.294 (GR) | Sanitary-Epidemiological and Public Health Department of Tashkent Region, Uzbekistan |
| **16** | **25** | EPI_ISL_1439597 | hCoV-19/Uzbekistan/Tashkent-CGB-25/2021 | 2020-12-07 | A.24 (S) | Sanitary-Epidemiological and Public Health Department of Tashkent Region, Uzbekistan |
| **17** | **27** | EPI_ISL_1477048 | hCoV-19/Uzbekistan/Tashkent-CGB-27/2020 | 2020-12-07 | A.24 (S) | Sanitary-Epidemiological and Public Health Department of Tashkent Region, Uzbekistan |
| **18** | **32** | EPI_ISL_1477049 | hCoV-19/Uzbekistan/Tashkent-CGB-32/2020 | 2020-12-07 | B.1.1.274 (GR) | Sanitary-Epidemiological and Public Health Department of Tashkent Region, Uzbekistan |
