# Supplementary material for: Profiling of the most reliable mutations from sequenced SARS-CoV-2 genomes scattered in Uzbekistan
Source: PLoS One. 2022 Mar 31;17(3):e0266417. doi: 10.1371/journal.pone.0266417 (PMC8970392; doi:10.1371/journal.pone.0266417)
Supplement: S3 Table — (DOCX) [file pone.0266417.s004.docx]

**S3 Table. Nucleotide mutations of the spike region of Uzbekistan SARS-CoV-2 sequences based on comparison to the reference sequence** (GenBank reference sequence accession number NC_045512.2).

| **#** | **Nucleotide position** | **Reference nucleotide** | **Sequenced nucleotide** | **Mutation type** | **Nucleotide change** | **Amino acid change (position)** |
| --- | --- | --- | --- | --- | --- | --- |
| 1 | 21850 | G | T | missense | G→T | E96D |
| 2 | 22335 | G | T | missense | G→T | W258 L |
| 3 | 24078 | A | G | missense | A→G | D839G |
| 4 | 24410 | G | C | missense | G→C | D950H |
| 5 | 23403 | A | G | missense | A→G | D614G |
| 6 | 23438 | G | T | missense | G→T | A626S |
| 7 | 23593 | G | T | missense | G→T | Q677H |
| 8 | 22020 | T | C | missense | T→C | M153T |
| 9 | 24872 | G | T | missense | G→T | V1104 L |
| 10 | 22478 | T | C | missense | T→C | F306 L |
| 11 | 22484 | G | T | missense | G→T | V308 L |
| 12 | 23634 | C | T | missense | C→T | S691F |
| 13 | 21724 | G | A | synonymous | G→A | L54 L |
| 14 | 25219 | T | G | synonymous | T→G | G1219G |
| 15 | 23503 | A | T | synonymous | A→T | A647A |
| 16 | 23758 | C | T | synonymous | C→T | T732T |
| 17 | 24023 | C | T | synonymous | C→T | L821 L |
| 18 | 24199 | G | T | synonymous | G→T | A879A |
| 19 | 24442 | C | T | synonymous | C→T | N960N |
| 20 | 29676 | A | G | downstream region | A→G | - |
| 21 | 29692 | G | T | downstream region | G→T | - |
| 22 | 29708 | C | T | downstream region | C→T | - |
| 23 | 29733 | C | T | downstream region | C→T | - |
| 24 | 29742 | G | A | downstream region | G→A | - |
| 25 | 29728 | TCACCGAGGCCACGCGGAGTACGATCGAG | - | downstream region | *4345_*4373del | - |
